# Dipeptide repeat proteins activate a heat shock response found in *C9ORF72*-ALS/FTLD patients

**DOI:** 10.1186/s40478-018-0555-8

**Published:** 2018-07-04

**Authors:** Daniel A. Mordes, Mercedes Prudencio, Lindsey D. Goodman, Joseph R. Klim, Rob Moccia, Francesco Limone, Olli Pietilainen, Kaitavjeet Chowdhary, Dennis W. Dickson, Rosa Rademakers, Nancy M. Bonini, Leonard Petrucelli, Kevin Eggan

**Affiliations:** 1 Department of Stem Cell and Regenerative Biology, Harvard University, Cambridge, MA 02138 USA; 2 Harvard Stem Cell Institute, Harvard University, Cambridge, MA 02138 USA; 3 Stanley Center for Psychiatric Research, Broad Institute of MIT and Harvard, Cambridge, MA 02142 USA; 4 Department of Pathology, Massachusetts General Hospital, Boston, MA 02114 USA; 5 Department of Neuroscience, Mayo Clinic, Jacksonville, FL 32224 USA; 6 Department of Biology, University of Pennsylvania, Philadelphia, PA 19104 USA; 7 Present address: Pfizer, Cambridge, MA 02139 USA

**Keywords:** Amyotrophic lateral sclerosis, *C9ORF72* repeat expansion, Dipeptide repeat proteins, Drosophila, Frontotemporal dementia, Frontotemporal lobar degeneration, HSF1, Heat shock response

## Abstract

A hexanucleotide (*GGGGCC*) repeat expansion in *C9ORF72* is the most common genetic contributor to amyotrophic lateral sclerosis (ALS) and frontotemporal lobar degeneration (FTLD). Reduced expression of the *C9ORF72* gene product has been proposed as a potential contributor to disease pathogenesis. Additionally, repetitive RNAs and dipeptide repeat proteins (DPRs), such as poly-GR, can be produced by this hexanucleotide expansion that disrupt a number of cellular processes, potentially contributing to neural degeneration. To better discern which of these mechanisms leads to disease-associated changes in patient brains, we analyzed gene expression data generated from the cortex and cerebellum. We found that transcripts encoding heat shock proteins (HSPs) regulated by the HSF1 transcription factor were significantly induced in *C9ORF72*-ALS/FTLD patients relative to both sporadic ALS/FTLD cases and controls. Treatment of human neurons with chemically synthesized DPRs was sufficient to activate a similar transcriptional response. Expression of *GGGGCC* repeats and also poly-GR in the brains of *Drosophila* lead to the upregulation of HSF1 and the same highly-conserved HSPs. Additionally, HSF1 was a modifier of poly-GR toxicity in *Drosophila*. Our results suggest that the expression of DPRs are associated with upregulation of HSF1 and activation of a heat shock response in *C9ORF72*-ALS/FTLD.

**Electronic supplementary material:**

The online version of this article (10.1186/s40478-018-0555-8) contains supplementary material, which is available to authorized users.

## Introduction

Amyotrophic lateral sclerosis (ALS) is an adult onset neurodegenerative disease characterized by the loss of upper and lower motor neurons and muscle atrophy. Patients become progressively paralyzed and develop difficulty speaking, swallowing, and eventually breathing. Survival is typically limited to 2–5 years from the time of onset, and current treatment options remain limited. About 90% of cases are seemingly “sporadic” without a family history of disease and about 10% are familial. Hundreds of distinct variants in more than a dozen genes, many of which act with high penetrance, can increase a person’s risk of developing ALS [46, 51].

The most common genetic contributor to ALS is a hexanucleotide (*GGGGCC*) repeat expansion within the first intron of *C9ORF72* [14, 45]. Carriers of the *C9ORF72* expansion can also present with frontotemporal dementia (FTD), which is characterized by frontotemporal lobar degeneration (FTLD) of the brain. In many cases, these initially diverse diagnoses can progress towards the inclusion of neurological features from each condition leading many to believe they are spectrums of the same disorder [52]. In addition, both diseases can be characterized by the presence of TDP-43 positive inclusions [37].

Three distinct mechanisms have been proposed for how the *C9ORF72* expansion contributes to the development of ALS and FTLD. First, *C9ORF72*-ALS brains display reduced abundance of *C9ORF72* transcripts, suggesting that a loss-of-function mechanism may contribute to disease [14]. Although complete loss of C9ORF72 in mice leads to fatal autoimmunity and changes in microglia, no obvious signs of neurodegeneration or neural dysfunction have yet been reported in these animals [8, 23, 39]. Second, mutant transcripts containing the *GGGGCC* repeats form intranuclear RNA foci that may sequester RNA binding proteins and lead to nucleolar stress [14, 20]. Finally, dipeptide repeat proteins (DPRs) were unexpectedly found to be translated from both sense and antisense transcripts containing these repeats [34]. Several DPRs are toxic when overexpressed in model systems [11, 26, 33, 55], and have been shown to affect diverse cellular pathways, including RNA processing and nucleocytoplasmic transport [51, 52, 56].

The transcriptional response that occurs in various brain regions in ALS and FTLD patients has the potential to provide useful insights into whether genetic subgroups of patients display common or divergent mechanisms, and for validating proposed mechanisms through which mutations act. Here, we explored RNA-sequencing data from *C9ORF72* and sporadic patients, and identified distinct transcriptional responses in these two patient classes. We validate a *C9ORF72*-specific transcriptional signature in a large patient cohort. Additionally, we find that similar transcriptional changes occur in human neurons treated with DPRs and in gain-of-function *Drosophila* models.

## Methods

### Bioinformatics

The processed gene expression count matrix of the brain-derived RNA-seq datasets from Prudencio et al. were obtained via GEO (GSE67196). The data was analyzed using the R library “edgeR” as described by Prudencio et al., with modifications as follows [41, 47]. Statistical inference was performed with two methods which we refer to as “double cut-off” and “FDR”. For the “double cut-off” method, as described by Prudencio et al., differentially expressed genes called by this approach had to pass two filters: one cut-off of absolute log2fold change > 2 and a second cut-off of unadjusted *p*-value < 0.05. For the “FDR” method, the false discovery rate was controlled using the Benjamini-Hochberg method [44] and all genes below a threshold FDR of 0.05 were considered to be significantly differentially expressed. Additionally, a generalized linear model (glmFit() in edgeR) was used to model the effect of gender rather than the exactTest() function, which resulted in slight differences in the number of differentially expressed genes found using the double cut-off method when compared to the original published analysis. Hypergeometric tests were used to compare sets of genes. Note that a pseudocount of 0.01 was used for plotting log2(CPM).

Protein-protein interaction networks were generated using GeNets hosted at the Broad Institute (apps.broadinstitute.org/genets) based on the InWeb network [28]. Associated gene ontology (GO) terms for biological process based on the GO Consortium were obtained with multiple testing correction for *p*-values using g:Profiler [43]. GO term clustering was performed with Revigo (reduce and visualize gene ontology, http://revigo.irb.hr/) [50] to support the identification of representative biological processes terms.

### Brain samples

Protocols were approved by the Mayo Clinic IRB and Ethics Committee on Human Experimentation. Informed consent for post-mortem tissue was obtained from all individuals or the appropriate next-of-kin. The diagnosis of ALS and/or FTLD was based on neurological and pathological examination and *C9ORF72* repeat expansion status was determined using repeat-primed PCR and the cohort was described in Prudencio et al., including TDP-43 pathology [42]. See Additional file 1: Table S1 for patient characteristics. For transcript measurements by quantitative RT-PCR on human brains, total RNA was extracted and 500 ng of RNA with RNA integrity values (RIN) higher than 7, measured by an Agilent Bioanalyzer, and was used for reverse transcription to synthesize cDNA as previously described [41]. Using a SYBR green assay (Life Technologies) samples were run in triplicate on an ABI Prism 7900HT Real-Time PCR System (Applied Biosystems). Relative mRNA expression of examined genes was normalized to GAPDH and RPLP0 values, the endogenous transcript controls. Primer sequences are provided in Additional file 2: Table S2. Statistical differences were calculated by one-way ANOVA followed by Dunn’s multiple comparison tests using GraphPad Prism. Associations between HSF1 and heat shock related transcripts were evaluated using a Spearman’s test of correlation.

### Neuron production and cell culture experiments

Neurons were generated from HuES-3-Hb9:GFP based on the following neuron differentiation protocol [6]. Human embryonic stem cells were cultured in mTeSR (Stemcell technologies) on matrigel (Corning)-coated plates. For motor neuron differentiation, the media was changed to 1:1 Neurobasal:DMEM/F12 (Life Technologies) supplemented with N2 (StemCell Technologies), B27 (Life technologies), Glutamax (Life Technologies), non-essential amino acids (Life technologies). For the first week, this neural media was supplemented with retinoic acid (Sigma Aldrich, 1 μM), smoothened agonist (SAG, DNSK, 1 μM), BMP inhibitor (LDN-193189, DNSK, 100 nM) and TGF-beta inhibitor (SB431542, DNSK, 10 μM). Then, for the second week, this neural media was supplemented with retinoic acid, smoothened agonist, GSK3-beta inhibitor (SU-5402, DNSK, 4 μM), and gamma-secretase inhibitor (DAPT, DNSK, 5uM). Upon completion of the differentiation protocol, cells were dissociated with accutase (Innovative Cell Technologies) to single cells and sorted via flow-cytometry for GFP-positive cells to yield GFP-positive neurons, which were plated on poly-D-lysine(Sigma Aldrich)/laminin(Life Technologies)-coated plates. Neurons were maintained in Neurobasal medium supplemented with N2, B27, Glutamax, non-essential amino acids, and neurotrophic factors (BDNF, GDNF, CNTF), and allowed to mature for two weeks before experiments with dipeptide repeat proteins (DPRs). Recombinant biotin-tagged DPRs, (each 20 amino acids in length (poly-GA, poly-GP, or poly-GR with 10 repeats or scrambled control poly-GAPR with 5 repeats) were synthesize by Anaspec with > 95% purity and dissolved in DMSO (Sigma). Following DPR treatment, RNA was extracted after 24 h via an RNeasy Minikit (Qiagen), and cDNA prepared with iScript (Bio-Rad). qRT-PCR reactions were performed with iTaq SYBR green (Bio-Rad) on a C1000 touch thermal cycler with CFX real-time system (Bio-rad). Relative expression was normalized to GAPDH. Primers were designed from the MGH PrimerBank and synthesized by IDT. Primer sequences are provided in Additional file 2: Table S2. Viability was measured with CellTiter-Glo (Promega) on a Cytation3 reader (Biotek). All cell lines tested negative for mycoplasma using the MycoAlert detection kit (Lonza LT07–518).

### Drosophila lines

Animals were raised and maintained at 18 °C on standard cornmeal-molasses food. The UAS-(G4C2)n transgenic models [9, 25], UAS-(GR)36 model [33], and the HSF overexpression (OE) mutant, HSF[+t8] [22], are previously defined. UAS-(GR)36, control, and mutant HSF[+t8] were obtained from Bloomington Drosophila Stock Center.

### qPCR in the adult fly nervous system

UAS-(G4C2)n or UAS-(GR)36 transgenes were driven by elavGS, a drug-inducible Gal4 driver that expresses only in neurons. Crosses were setup and maintained at 24 °C. Female progeny with the desired genotype were collected and matured to 1-3d before being transferred to vials containing 40μg/ml of RU486. Animals were aged on RU486-infused food 16d while being flipped onto fresh drug-infused food every 2-days. Total RNA was collected from heads of frozen animals using Trizol, converted to cDNA using random primers, and analyzed by qPCR using SYBR Green. All primers were previously developed with the exception of dHSF1, dHSP70, dBAG3, dStip1, dFkbp4 (Fkbp59), and dChordc1 [5, 12]. Data was normalized to the housekeeping gene, RP49 [17]. Primer sequences are provided in Additional file 2: Table S2. Full genotypes for (G4C2)n are as follows: w1118/yw;; UAS-(G4C2)n, elavGS/+. (GR)36 animals, w1118/yw; UAS-(GR)36/+; elavGS/+, were compared to controls, w1118/yw;;. For analysis of HSF mutant expression, briefly, male HSF OE mutant flies were crossed to w1118 virgin females and maintained at 24 °C. Male progeny were collected and aged to 5d before analysis. Full genotype: w1118;; HSF[+t8]/+. Control w1118 males were maintained and aged in parallel.

### External eye analysis

Scoring of the external eye phenotype for (G4C2)49 was done using a 0–8 scale previously defined where 0 = WT eye and 8 = lethality (extreme toxicity) [25]. (G4C2)49 expression causes an average degenerative score of 4–5 across multiple studies. Scoring of the external eye phenotype for (GR)36 was done using a 0–11 scale where 0 = WT eye and 11 = lethality (extreme toxicity) (Additional file 3: Figure S5). (GR)36 expression causes an average degenerative score of 5–6 across multiple studies.

For optimal eye phenotypes, crosses for (G4C2)n were setup and maintained at 24 °C and (GR)36 at 21 °C. Male progeny with the desired genotype were collected daily and matured to 1-2d before imaging on a Leica Apo16 microscope. Severity of the external eye phenotype was determined post-imaging while looking for changes in red pigmentation, ommatidial organization, and eye size. Full genotypes for (G4C2)n are as follows: “Control” = w1118;; UAS-(G4C2)n, Gmr-Gal4/+ and “HSF OE” = w1118;; UAS-(G4C2)n, Gmr-Gal4/HSF[+t8]. Full genotypes for (GR)36 are as follows: “Control” = w1118; UAS-(GR)36/+; Gmr-Gal4/+ and “HSF OE” = w1118; UAS-(GR)36/+; Gmr-Gal4/HSF[+t8].

### Drosophila beta-galactosidase western blots

Western blots are as previously described [25].

## Results

### Identification of a *C9ORF72*-associated transcriptional signature in patient brain samples

**Fig. 1:**
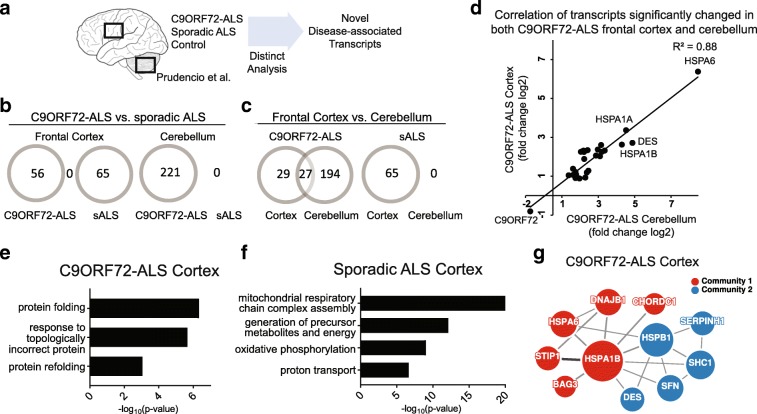
Identification of specific cellular pathways perturbed in sporadic ALS and C9ORF72-ALS **a** Diagram of RNA-seq datasets obtained from the frontal cortex and cerebellum by Prudencio et al. **b** Comparison of the significant (FDR < 0.05) differentially expressed transcripts in C9ORF72-ALS (C9-ALS) and sporadic ALS (sALS). Note, there were no common transcripts between C9ORF72-ALS and sporadic ALS in either brain region. **c** Comparison of the differentially expressed transcripts by brain region. **d** Correlation of the fold change (log2) of changed transcripts in C9ORF72-ALS that were common to both brain regions (Spearman’s R^2^) **e**, **f** Gene ontology (GO) analysis revealed cellular processes affected in C9ORF72-ALS and sALS. **g** Protein-protein interaction analysis of proteins encoded by the transcripts changed in C9ORF72-ALS revealed a protein chaperone network

There remains much to be learned concerning the mechanisms by which the repeat expansion in *C9ORF72* contributes to ALS and FTLD. Recently, RNA-sequencing datasets were generated from the frontal cortex as well as the cerebellum of sporadic ALS cases, *C9ORF72*-ALS cases and controls [41]. In Prudencio et al., a “double-cutoff method” was used for identifying genes whose expression was significantly changed in each class of ALS patient relative to controls (methods). Although such methods are useful for identifying changes in gene expression, they tend to be more sensitive to large fold-changes in less abundant transcripts, while modest fold-changes in abundant transcripts may go undetected (Additional file 4: Figure S1) [3, 29]. We reasoned that further analyses of these data might provide new insights into the disease mechanisms acting in *C9ORF72* and sporadic patients, respectively. Utilizing a false discovery rate (FDR) threshold of 5%, we sought to identify changes in abundantly-expressed transcripts and found 56 transcripts that were differentially expressed between *C9ORF72*-ALS cortex and controls at this confidence interval (Additional file 4: Figure S1, methods). Comparison of sporadic ALS patient and control cortex with these same metrics identified 65 differentially expressed transcripts, most (61) of which were downregulated. Consistent with the previous report that sporadic and *C9ORF72* ALS patients display distinct transcriptional signatures relative to controls, we found no overlap in the identity of transcripts that we identified as differentially expressed in the cortex of sporadic ALS and *C9ORF72* ALS patient classes (Fig. 1b) [41]. However, the majority of the genes we had found as likely to be differentially expressed in sporadic and *C9ORF72*-ALS patients had not been previously identified [41], validating the importance of reanalyzing these sequencing data using the methods we employed (Additional file 5: Table S3, Additional file 4: Figure S1, see Methods).

In *C9ORF72-*ALS, the cortex is characterized by distinct p62-positive DPR neuronal inclusions and the cerebellum contains abundant DPR inclusions [1, 30, 34, 41]. Hence, we reasoned that identifying transcripts with expression changes shared in both the frontal cortex and the cerebellum might lead us to genes and pathways that were reproducibly induced by the *C9ORF72* repeat expansion. Strikingly, 27 of the 56 transcripts differentially expressed in the *C9ORF72*-ALS cortex were also significantly changed in the *C9ORF72-*ALS cerebellum (*p* = 2.93*10^− 40^; Fig. 1c). Comparison of the fold expression changes in these 27 transcripts between these two regions in *C9ORF72*-ALS brains revealed a strong positive correlation (R^2^ = 0.88). Notably, we identified increased abundance for 26 of these 27 transcripts in both brain regions relative to controls (Fig. 1d). The one exception was the *C9ORF72* transcript itself which showed reduced abundance (57% cortex, FDR = 0.0169; 42% cerebellum, FDR = 2.75*10^− 5^), in agreement with previous studies of patient brains [14, 52, 53]. In contrast to a prior analysis, we detected no significant transcriptional changes between the cerebellum of sporadic ALS cases and controls (Fig. 1b), consistent with this region being histologically unremarkable in sporadic cases [2].

To determine if the transcripts significantly changed in *C9ORF72* and sporadic ALS cortex pointed to particular pathways that might be responding to disease processes, we carried out gene ontology (GO) analysis. Transcripts identified in *C9ORF72*-ALS were significantly associated with response to topologically incorrect proteins (*p* = 2.13*10^− 6^) and protein folding (*p* = 4.57*10^− 7^) (Fig. 1e). In contrast, differentially expressed transcripts detected in sporadic ALS were associated with functions in the mitochondrial respiratory chain complex assembly (*p* = 8.53*10^− 21^) and related terms (Fig. 1f), and included 9 members of the NADH dehydrogenase (complex I) enzyme and 6 components of cytochrome oxidase C (complex IV) (Additional file 6: Figure S2A). These findings suggest that the transcriptional responses in the *C9ORF72* and sporadic ALS cortex might be reflective of changes in protein and mitochondrial homeostasis, respectively. We also asked whether any of the *C9ORF72*-associated transcripts encoded proteins that interact in particular complexes. Using the InWeb protein-protein interaction network [28], analysis of the 56 differentially expressed transcripts from *C9ORF72* cortex identified an interaction network involving heat shock proteins (HSPs) and protein chaperones, with HSPA1B (HSP70) and HSPB1 (HSP27) at its hubs (Fig. 1g). Examination of protein interactions from the 221 transcripts differentially expressed in the *C9ORF72* cerebellum revealed a similar and expanded network of more than 80 interactors that was centered on the same core HSPs (Additional file 6: Figure S2B).

### Activation of the HSF1 pathway in *C9ORF72*-ALS/FTLD

A well-established regulator of HSP and protein chaperone expression is the transcription factor heat shock factor 1 (HSF1) [57]. Hence, we wondered if the transcriptional response we observed in the *C9ORF72* brain might be at least in part mediated by activation of HSF1. To explore this possibility, we turned our attention to established HSF1 target genes previously identified by ChIP-seq and genome-wide methods [31, 32, 49]. Consistent with the notion that a portion of the response in *C9ORF72* patient brain was mediated by HSF1, 13 of the 27 transcripts identified as significantly changed in both the cerebellum and frontal cortex were among 812 genes bound by HSF1 after heat shock treatment across three human cell lines (*p* = 1.22*10^− 12^) [32], including several HSPs shown to be upregulated in the initial small *C9ORF72*-ALS cohort [41].

**Fig. 2:**
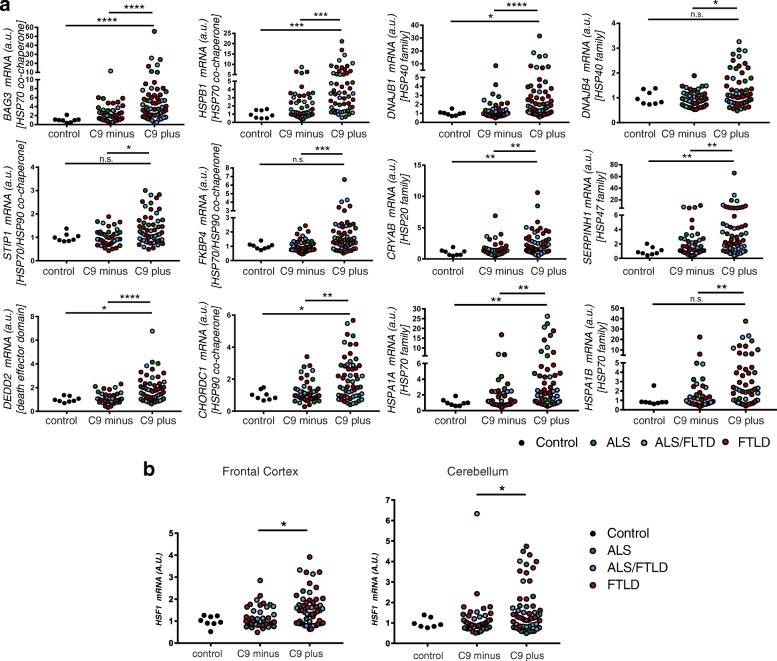
Activation of HSF1 in C9ORF72-ALS, FTLD, and combined ALS/FTLD patients. **a** Quantitative real-time PCR (qRT-PCR) for HSF1 target genes in the frontal cortex of sporadic and C9ORF72-associated disease (*n* = 56 C9ORF72-ALS/FTLD, *n* = 46 sporadic ALS/FTLD, *n* = 9 controls) (one-way ANOVA with Bonferonni post-hoc test for multiple comparisons, *p < 0.05, **p < 0.01, ***p < 0.001). No significant changes were detected between the sporadic cases and controls (mean values for each gene are provided in Additional file: 3 Table S3). **b** qRT-PCR for HSF1 in the frontal cortex and cerebellum of these same cases

As a next step towards investigating whether activation of the HSF1 might be responsible for the induction of these genes in *C9ORF72* patients, we used quantitative RT-PCR to measure the transcript abundance of *HSF1* and 11 of these conserved HSP-associated transcripts in a much larger patient cohort that also included patients diagnosed with FTLD and both ALS and FTLD (*n* = 56 *C9ORF72*-ALS/FTLD, *n* = 46 sporadic ALS/FTLD, *n* = 8 controls). In the frontal cortex, expression of each of these 11 HSF1 target genes was significantly increased in the *C9ORF72*-ALS/FTLD cohort relative to controls (*p* < 0.05 or lower for each gene) and to sporadic cases (*p* < 0.01 or lower) (Fig. 2a, Additional file 7: Table S4). Next, we extended our expression analyses of these 11 HSF1 targets to the cerebellum, and again found that the abundance of each transcript gene was significantly elevated in *C9ORF72*-ALS/FTLD relative to both controls and sporadic ALS cases (Additional file 8: Figure S3A, Additional file 7: Table S4). For example, we found a significant, two orders of magnitude induction of the HSP70 transcript *HSPA1B* in *C9ORF72*-ALS/FTLD cases relative to controls. To investigate if the larger number of genes initially detected only in the *C9ORF72* cerebellum might also be reflective of a heat shock response, we examined another HSF1 target gene *CRYAB* and found it was significantly upregulated in both *C9ORF72* brain regions in this larger patient cohort (Fig. 2a, Additional file 8: Figure S3A).

Evaluation of *HSF1* expression in these same samples demonstrated that it was significantly more abundant in both the cortex and cerebellum relative to sporadic ALS cases (*P* < 0.05) (Fig. 2b). We found a strong and consistent correlation between the levels of *HSF1* and each of these *C9ORF72*-chaperome transcripts in both brain regions (*p* < 0.0001 for each gene, Additional file 8: Figure S3B). For instance, the relationship between the transcript levels of *HSF1* and *HSPB1* yielded an R^2^ value of 0.73 (95% CI 0.63–0.81) in the cortex and 0.65 (95% CI 0.52–0.75) in the cerebellum. Taken together, these findings indicate that HSF1 is activated in *C9ORF72*-ALS and FTLD patient brains and suggests that it is regulating the expression of the HSPs we found to be induced there.

### DPRs are sufficient to induce a *C9ORF72-*associated transcriptional changes

**Fig. 3:**
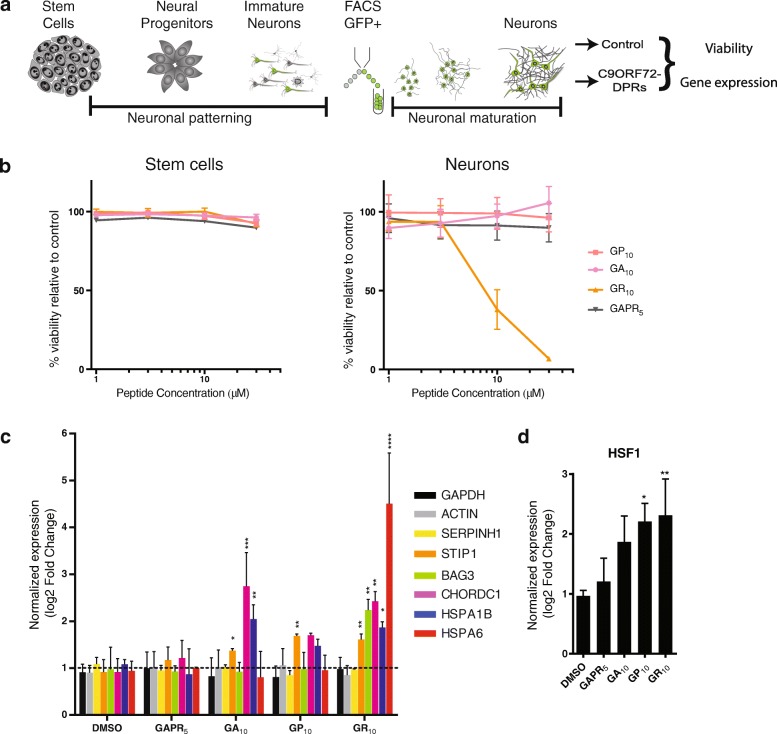
DPRs induce expression of C9ORF72 signature transcripts in human neurons. **a** Diagram of generation of human neurons from stem cells. **b** Viability dose response curve of human stem cells and stem-cell derived neurons exposed to various DPRs (*n* = 6). **c, d** qRT-PCR of C9ORF72-chaperome transcripts (**c**) and HSF1 (**d**) in human stem cell-derived motor neurons following treatment with DPRs (poly-GA, poly-GP, poly-GR) or a scrambled poly-GAPR (5 uM for 24 h) normalized to control (DMSO-treated) neurons (mean ± SD, *n* = 3, one-way ANOVA with Dunnett’s post-hoc test for upregulated genes in DPR-treated neurons compared to control, * *p* < 0.05, ** *p* < 0.01, *** *p* < 0.001, **** *p* < 0.0001)

The *C9ORF72 GGGGCC* repeat expansion is translated from both sense and anti-sense transcripts through non-ATG translation to generate 5 distinct dipeptide repeat proteins (DPRs), e.g. poly-glycine-arginine (poly-GR) [18, 34, 52]. We wondered if DPRs alone were sufficient to induce the upregulation of C9ORF72 signature transcripts. Therefore, we tested the effects of synthetic DPRs in human stem cell-derived neurons [26]. GP_10_, GA_10_ and a scrambled GAPR_5_ control were not acutely toxic to the parental human stem cell line or stem-cell derived neurons. In contrast poly-GR_10_ resulted in a dose-dependent decrease in the viability of stem cell-derived neurons, but not the stem cell from which they were produced (Fig. 3b). In human neurons, we found that both poly-GA and poly-GR led to the significant upregulation of *HSPA1B* (*p* < 0.01), as well as additional C9ORF72 signature transcripts (Fig. 3c). Given that poly-GA is not associated with decreased viability in these conditions, this suggests that the observed transcriptional changes are not simply a consequence of general neuronal toxicity. There was a strong correlation (R^2^ = 0.58) between the degree of induction of these transcripts in human neurons by poly-GR and the changes present specifically in *C9ORF72* brains. Upon measuring *HSF1*, there was a trend for increased levels with poly-GA and poly-GP, and the greatest increase was again observed with poly-GR (Fig. 3d). These findings support the notion that gain-of-function effects from DPRs are sufficient to induce HSF1 target genes that are upregulated in *C9ORF72*-associated disease.

### Detection of *C9ORF72-*associated transcriptional changes in gain-of-function *Drosophila* models

**Fig. 4:**
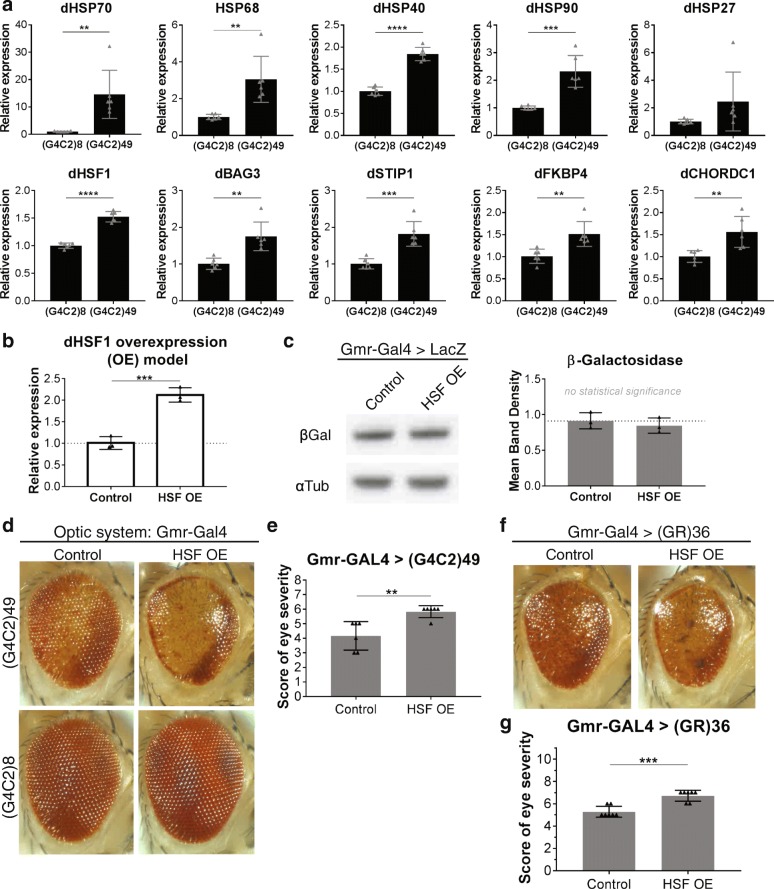
**a** Control UAS-(G4C2)8 and expanded UAS-(G4C2)49 transgenes were expressed in the adult fly nervous system using the drug-inducible Gal4 driver, elavGS, for 16d. Quantitative PCR (qPCR) analysis of endogenous dHSF1 and HSF1-regulated genes revealed significant upregulation with (G4C2)49 expression compared to (G4C2)8 controls. Differences in expression are likely underestimated as the analyses include neuronal and non-neuronal tissue while (G4C2)n was expressed only in neurons. **b** qPCR analysis of a dHSF1 overexpression mutant fly line shows endogenous HSF is upregulated approximately 2-fold in mutant flies compared to control. **c** Western immunoblot analysis of expression of a control UAS-LacZ transgene confirmed that the HSF OE mutant did not affect the Gal4/UAS expression system. **d** (G4C2)49 was expressed in the optic system of control animals or HSF OE animals using Gmr-Gal4. (G4C2)49 causes toxicity seen by pigment loss, reduced eye size, and disruptions in the normal ommatidial organization. In HSF OE animals, toxicity of (G4C2)49 is enhanced – animals have increased pigment loss, increased disruption of ommatidial organization, and further reduced eye size. Expression of control (G4C2)8 in the fly optic system (Gmr-Gal4) of control and HSF OE animals does not affect the external eye. **e** Quantification of the external eye degenerative phenotype caused by (G4C2)49 expression shows enhancement in HSF OE animals versus control animals to be consistent and statistically significant (*n* = 6). Animals received a score between 0 (WT eye) and 8 (lethality caused by extreme degeneration in the optic system). (G4C2)49 expression causes an average score of 4 in controls. **f** Gmr-GAL4 driven expression of (GR)36 shows toxicity in control scenarios like (G4C2)49. HSF OE in these animals also causes enhanced toxicity (increased pigment loss, increased disruption of ommatidial organization, and reduced eye size) **g** Quantification of the external eye degenerative phenotype caused by (GR)36 expression shows enhancement in HSF OE animals versus control animals to be consistent and statistically significant (*n* = 7). Animals received a score between 0 (WT eye) and 11 (lethality caused by extreme degeneration in the optic system) while (GR)36 causes an average score of 5 in controls. (All plots: mean +/− SD, unpaired, student’s t-test, *p < 0.05, **p < 0.01, ***p < 0.001, ****p < 0.0001)

To test for correlations in DPR production and altered HSF1 target gene expression in vivo, we evaluated a *Drosophila* gain-of-function transgenic model engineered to express 49 pure *GGGGCC* repeats driven by a drug-inducible neuronal-specific *ElavGS-GAL4* driver [25, 33]. Fly models expressing toxic GGGGCC repeats produce DPRs and RNA foci [16, 33, 54]. We found significant increased expression of the *Drosophila* orthologs of conserved *C9ORF72*-associated HSPs and protein chaperones in flies expressing 49 repeats in neurons compared to controls (Fig. 4a). Upregulation of HSF1-associated genes was observed in the absence of significant animal death, arguing that the effect is specific to expression of (*GGGGCC*)_49_. Note that these expression changes are likely to be an underestimation of the actual changes caused by the repeats in vivo since (*GGGGCC*)_49_ was only expressed in neurons while gene expression changes were assayed using whole heads, including non-neuronal tissue. Additionally, we detected an increase in HSF1 expression similar to that observed in *C9ORF72* patient brains (Fig. 4a). This demonstrates that at least part of the transcriptional response to the *C9ORF72* repeat expansion is conserved in *Drosophila*, and is consistent with gain-of-function effects of the *C9ORF72* repeat expansion driving the expression of HSF1 target genes.

Activation of the HSF1 pathway has been proposed to be protective in several neurodegenerative diseases associated with protein aggregation as a means to combat the cellular effects of toxic proteins [35]. Given that we observed an HSF1 heat shock response in *C9ORF72* patients and model systems, we wondered whether HSF1 may be a potential modifier of *C9ORF72* gain-of-function toxicity. To investigate this idea, we selected a fly line harboring an additional allele of the *Drosophila HSF1* ortholog (*dHSF*1) [22]. We confirmed increased *dHSF1* expression in this line and noted that it was comparable to the relative increase in *dHSF1* expression observed in response to the *GGGGCC* repeat expansion (Fig. 4b). The presence of additional *dHSF1* did not affect the expression of a control *LacZ* transgene (Fig. 4c). We next asked if this increase in *dHSF1* would have an effect on *GGGGCC-*mediated toxicity and used the *Gmr-Gal4* driver to specifically express the repeats in the fly optic system to assess the effect on the eye. Consistent with prior observations, *GGGGCC*_49_ expression in the eye during development led to generation of animals with eye degeneration and disruption of the highly regular ommatidial structure, reduced eye size, and loss of pigment (Fig. 4d) [25, 33]. *dHSF1* upregulation by itself did not affect eye structure in the presence of a control *GGGGCC*_8_ (Fig. 4d). Surprisingly, we found that *GGGGCC*_49_-induced toxicity in the external eye was enhanced in the presence of *dHSF1* overexpression (Fig. 4d, e).

Among the repeat expansion encoded DPRs, arginine-rich DPRs are particularly toxic in model systems, including *Drosophila* [33]. Given that the expression of *GGGGCC*_49_ is associated with the production of both DPRs and potentially toxic RNA, we assayed the transcriptional effects of poly-GR in vivo. There was significant upregulation of *dHSF1* and many HSF1-regulated transcripts in *Drosophila* expressing a poly-GR_100_ transgene in neurons compared to non-transgenic controls (Additional file 9: Figure S4). We additionally tested the effects of modulating *dHSF1* levels in the optic system of poly-GR *Drosophila* again using *Gmr-GAL4* to drive transgene expression. We observed exacerbation of poly-GR_36_ external eye toxicity in the presence of *dHSF1* upregulation (Fig. 4h, j). These results argue that the changes in toxicity caused by added *dHSF1* in the *GGGGCC*_49_ model is in part due to the effects of GR-dipeptide. Taken together, these findings suggest that augmentation of HSF1 activity may enhance DPR-mediated toxicity in *Drosophila*.

## Discussion

In this study, we have identified novel differentially expressed transcripts in *C9ORF72-*ALS based on analysis of two brain regions compared to controls. Every *C9ORF72-*associated transcript was not significantly altered in sporadic ALS, suggesting that the observed changes in this set of transcripts are not just an indicator of neuronal loss but rather reflective of *C9ORF72*-specific pathogenesis. Furthermore, we validated our *C9ORF72* transcriptional signature in a large ALS/FTLD patient cohort and gain-of-function models.

Our findings specifically link activation of the HSF1 pathway to *C9ORF72-*ALS/FTLD. The HSF1 pathway is highly conserved from budding yeast to mammals and is an important mediator of the compensatory response to disruptions in proteostasis, such as heat shock [49]. Impairment of HSF1 activity and loss of protein chaperone function have been reported to occur with ageing and in the setting of age-related neurodegeneration [7, 21, 35]. For instance, in models of poly-glutamine repeat-associated Huntington disease decreased expression of HSF1 target genes is observed and may contribute to protein aggregation [10]. Likewise, decreased expression of a particular set of protein chaperones, including HSP90, occurs in Alzheimer disease and Parkinson disease [7]. In *C9ORF72*-ALS/FTLD, we found robust increased expression of a family of protein chaperones and co-chaperones, consistent with activation of a heat shock response in this particular disease. Our study may provide the first evidence of increased, rather than impaired, activity of HSF1 based on human brain samples for a specific neurodegenerative disease. In addition, HSF1 is generally not thought to be regulated at the transcriptional level in the context of neurodegeneration. We found upregulation of HSF1 itself in *C9ORF72*-ALS/FTLD and a strong correlation between levels of HSF1 and its target genes. It will be important to examine transcriptional changes in HSF1 in post-mortem brain samples of additional neurodegenerative diseases.

Prior studies in model systems have suggested that HSF1 is a protective factor that helps neurons cope with cellular stress associated with misfolded proteins and protein aggregates [19]. Unexpectedly, we observed that having additional HSF1 in the developing eye in two *Drosophila* models of *C9ORF72-*ALS/FTLD was not beneficial. Additional studies in these *Drosophila* models that characterize the functional consequences of the loss of individual HSF1-associated protein chaperones and HSPs may be useful to further dissect the relationship between this pathway and *C9ORF72*-related pathology. Pharmacological activation of HSF1 has been proposed as a therapeutic strategy to enhance protein chaperone function and neuronal survival in neurodegenerative disease [36]. For instance, arimoclomol, which may act to enhance HSF1-pathway activation, has been shown to delay disease progression in an SOD1 overexpression mouse model [24]. A phase II clinical trial for arimoclomol was recently conducted for a subtype of familial ALS associated with mutations in SOD1 and was found to be well-tolerated [4]. Our findings suggest that additional pre-clinical studies may be warranted if this strategy is applied to other forms of ALS, especially the most common type of ALS, *C9ORF72*-ALS, and associated dementias. Additionally, the transcriptional differences present among distinct cohorts of ALS/FTLD patients re-emphasizes the potential importance of patient stratification by genotype for future clinical trials.

Several studies have aimed to identify the specific transcriptional changes and pathways affected by the *C9ORF72* repeat expansion in patient-based cellular models, including iPSC-derived neurons, with little concordance among them [13, 15, 27, 48]. Gene expression changes have also been explored in a few animal models for this disease. In a loss-of-function mouse model lacking both copies of *C9ORF72*, transcriptional analysis of the spinal cord from *C9ORF72−/−* animals revealed significant changes in several pathways related to inflammation [39]. On the other hand, gain-of-function bacterial artificial chromosome (BAC) transgenic mouse models harboring the human *C9ORF72* repeat expansion have been generated with varying phenotypes and transcriptional changes. In one model containing exons 1–6 of human *C9ORF72* with approximately 500 hexanucleotide repeats, no significant changes in the transcriptome of the frontal cortex at 6 months of age were reported [40]. In another BAC mouse model with 100–1000 repeats, immunomodulatory and extracellular matrix pathways were identified as being altered in the frontal cortex also at 6 months of age [38]. Although both of these BAC mouse models exhibit DPR inclusions in the nervous system that increase with age, evidence of neurodegeneration was not observed. One possibility is that DPRs did not reach sufficient levels in these models at the examined time points to induce neurodegeneration or the transcriptional changes described herein. Indeed, robust expression of DPRs using an adeno-associated viral vector with 66 repeats was sufficient to induce DPR aggregates, TDP-43-positive inclusions, neuronal loss, and behavioral deficits in mice [11]. However, gene expression studies have not yet been performed in this viral mouse model.

Using two gain-of-function *Drosophila* models, we found upregulation of many *Drosophila* orthologs of the same genes that were upregulated in *C9ORF72* patient brains. This is consistent with the notion that more potent expression of DPRs in models is essential to recapitulate *C9ORF72* transcriptional changes and disease phenotypes. Our approach and findings starting with unbiased transcriptional analysis of patient samples may be useful for the characterization and assessment of existing and new models employed to study *C9ORF72* disease.

Based on our findings, we propose the following model. The presence of the *C9ORF72* repeat expansions results in the production of various toxic DPRs. In early life, neurons can degrade DPRs or perhaps sequester them into protective p62-positive inclusions. With aging, there is a decreased capacity of neurons to maintain proteostasis, and environmental insults may be associated with additional proteotoxic stress. This leads to the gradual accumulation of DPRs and the activation of a heat shock response to increase protein chaperones, perhaps in an attempt to refold inherently unstructured DPRs. However, increased HSF1 activity may actually contribute to DPR-dependent toxicity. One possibility is that the resulting increased levels of protein chaperones may promote the solubility or the stability of toxic DPRs. This model could partially explain the variable disease penetrance and expressivity by which the C9ORF72 repeat expansion acts to cause ALS and/or FTLD. It could be that natural human variation in the HSF1 response influences when and where the repeat expansion results in neurodegeneration.

## Conclusions

In summary, we have identified specific gene expression changes in *C9ORF72* disease that are consistent with the activation of a HSF1-associated transcriptional response. We found that the expression levels of HSF1 and protein chaperones are increased in *C9ORF72-*ALS/FTLD patients and in gain-of-function model systems. Our results suggest that DPRs encoded by the *C9ORF72* hexanucleotide repeat expansion are sufficient to lead to the upregulation of HSF1 and its target genes. The effects of the HSF1 pathway on *C9ORF72* pathogenesis in models of disease that express DPRs warrants further investigation.

## Additional files

Additional file 1: **Table S1.** Characteristics of patient cohort for brain samples used in qPCR analysis. (PDF 154 kb)

Additional file 2: **Table S2.** Primer sequences for PCR. (PDF 167 kb)

Additional file 3: **Figure S5.** External eye quantification scale for (GR)36 animals. For quantification of the enhancement effects of increased expression of HSF in the eye, (GR)36 animals received a score between 0 (normal eye) and 11 (extreme toxicity causing lethality). Across multiple studies control Gmr-GAL4 > (GR)36 animals receive a score between 5 and 6. (PDF 1969 kb)

Additional file 4: **Figure S1.** Bioinformatic method comparison for gene expression analysis of C9ORF72-associated ALS and sporadic ALS (sALS) in the frontal cortex and cerebellum. (a-d) Gene density plots comparing the expression levels of differentially expressed transcripts as determined by the prior double cut-off method (|log2 fold change| ≥ 2 and *p*-value < 0.05) and the FDR method (FDR < 0.05), which was used in Fig. 1 and subsequent analysis. Note, no significant changes were detected in the sALS cerebellum using with FDR method. (e) Venn diagrams demonstrating considerable differences in the designated disease-associated transcripts between these bioinformatics methods in both brain regions. (f) Table of the number of differentially expressed transcripts as determined by Prudencio et al., the double cut-off method and the FDR method used here. (PDF 216 kb)

Additional file 5: **Table S3.** Genes significantly changes in RNA-seq analysis from ALS patients. (XLSX 52 kb)

Additional file 6: **Figure S2.** Gene networks in C9ORF72-ALS and sporadic ALS. (a) Protein-protein interaction network derived from differentially expressed transcripts in C9ORF72-ALS cerebellum. Those transcripts that are differentially expressed in both the frontal cortex and the cerebellum in C9ORF72-ALS are highlighted by dashed yellow circles, and predominantly consist of heat shock proteins and protein chaperones. (b) Protein-protein interaction network derived from differentially expressed transcripts in the sporadic ALS cortex. (PDF 164 kb)

Additional file 7: **Table S4.** Values from qPCR analysis in brain samples. (PDF 178 kb)

Additional file 8: **Figure S3.** Activation of HSF1 in C9ORF72-ALS, FTLD, and combined ALS/FTLD patients. (a) Quantitative real-time PCR (qRT-PCR) for HSF1 target genes in the cerebellum of sporadic and C9ORF72-associated disease (*n* = 56 *C9ORF72-*ALS/FTLD, *n* = 42 sporadic ALS/FTLD, *n* = 7 controls) (one-way ANOVA with Bonferonni *post-hoc* test for multiple comparisons * *p* < 0.05, ** *p* < 0.01, *** *p* < 0.001. Note, no significant changes were detected between the sporadic cases and controls. (b) Correlation of HSF1 levels and HSF1 target gene levels in the frontal cortex and cerebellum in C9ORF72-ALS/FTLD. Spearman’s R^2^ values are plotted for each target gene, error bars denote 95% confidence interval, *p*-value < 0.0001 in all cases. (PDF 209 kb)

Additional file 9: **Figure S4.** poly-GR expression results in the upregulation of heat shock response genes and dHSF1 in the adult fly nervous system. UAS-(GR)36 was expressed in the adult fly nervous system using the drug-inducible Gal4 driver, elavGS, for 16d. qPCR analysis of endogenous HSF1-regulated genes and dHSF1 revealed significant upregulation of the *Drosophila* orthologs of many of the genes identified in patient studies, suggesting that poly-GR is contributing to the altered transcriptome in C9ORF72-ALS/FTLD patients. Control animals did not express a transgene. Differences in expression are likely underestimated as the analyses include neuronal and non-neuronal tissue while (GR)36 was expressed only in neurons. (*n* = 6, mean +/− SD, unpaired, student’s t-tests, *p-value* * < 0.05, ** < 0.01). (PDF 389 kb)
