# Supplementary material for: Dipeptide repeat proteins activate a heat shock response found in C9ORF72-ALS/FTLD patients
Source: Acta Neuropathol Commun. 2018 Jul 4;6:55. doi: 10.1186/s40478-018-0555-8 (PMC6031111; doi:10.1186/s40478-018-0555-8)
Supplement: Supplementary file 1 — Table S1. Characteristics of patient cohort for brain samples used in qPCR analysis. (PDF 154 kb) [file 40478_2018_555_MOESM1_ESM.pdf]

| | FRONTAL CORTEX | | | |
|---|---|---|---|---|
| | # cases (sex) | Average age onset +/- SEM | Average age death +/- SEM | Average survival +/- SEM |
| C9 minus | 46 (24F, 22M) | 64.91 ± 1.61 (N=41) | 72.39 ± 1.78 (N=46) | 6.44 ± 0.86 (N=41) |
| ALS | 17 (9F, 8M) | 58.26 ± 2.55 (N=14) | 64.99 ± 2.40 (N=17) | 4.44 ± 1.36 (N=14) |
| ALS/FTD | 13 (7F, 6M) | 64.17 ± 2.33 (N=13) | 69.31 ± 2.52 (N=13) | 5.14 ± 1.56 (N=13) |
| FTD | 16 (8F, 8M) | 72.23 ± 2.16 (N=14) | 82.76 ± 2.26 (N=16) | 9.64 ± 1.23 (N=14) |
| C9 plus | 56 (24F, 32M) | 60.57 ± 1.30 (N=52) | 66.69 ± 1.44 (N=55) | 5.78 ± 0.51 (N=52) |
| ALS | 13 (9F, 4M) | 55.56 ± 2.75 (N=13) | 58.11 ± 2.71 (N=13) | 2.56 ± 0.42 (N=13) |
| ALS/FTD | 19 (8F, 11M) | 59.40 ± 1.67 (N=17) | 64.27 ± 1.75 (N=18) | 5.03 ± 0.66 (N=17) |
| FTD | 24 (7F, 17M) | 64.43 ± 1.99 (N=22) | 73.15 ± 1.90 (N=24) | 8.26 ± 0.77 (N=22) |
| Controls | 8 (3F, 5M) | N/A | 73.43 ± 2.362 (N=8) | N/A |

| | CEREBELLUM | | | |
|---|---|---|---|---|
| | # cases (sex) | Average age onset +/- SEM | Average age death +/- SEM | Average survival +/- SEM |
| C9 minus | 42 (22F, 20M) | 65.18 ± 1.71 (N=38) | 72.58 ± 1.91 (N=42) | 6.57 ± 0.92 (N=38) |
| ALS | 16 (8F, 8M) | 58.29 ± 2.75 (N=13) | 63.97 ± 2.41 (N=15) | 4.45 ± 1.25 (N=13) |
| ALS/FTD | 11 (6F, 5M) | 64.36 ± 2.62 (N=11) | 69.51 ± 2.93 (N=11) | 5.16 ± 1.83 (N=11) |
| FTD | 15 (8F, 7M) | 72.23 ± 2.16 (N=14) | 82.76 ± 2.26 (N=16) | 9.64 ± 1.23 (N=14) |
| C9 plus | 56 (24F, 32M) | 60.57 ± 1.30 (N=52) | 66.69 ± 1.44 (N=55) | 5.78 ± 0.51 (N=52) |
| ALS | 13 (9F, 4M) | 55.56 ± 2.75 (N=13) | 58.11 ± 2.71 (N=13) | 2.56 ± 0.42 (N=13) |
| ALS/FTD | 19 (8F, 11M) | 59.40 ± 1.67 (N=17) | 64.27 ± 1.75 (N=18) | 5.03 ± 0.66 (N=17) |
| FTD | 24 (7F, 17M) | 64.43 ± 1.99 (N=22) | 73.15 ± 1.90 (N=24) | 8.26 ± 0.77 (N=22) |
| Controls | 7 (3F, 4M) | N/A | 72.76 ± 2.62 (N=7) | N/A |

**Table S1: Patient cohort characteristics of brain samples used for qRT-PCR.**
