# Supplementary material for: Dipeptide repeat proteins activate a heat shock response found in C9ORF72-ALS/FTLD patients
Source: Acta Neuropathol Commun. 2018 Jul 4;6:55. doi: 10.1186/s40478-018-0555-8 (PMC6031111; doi:10.1186/s40478-018-0555-8)
Supplement: Supplementary file 2 — Table S2. Primer sequences for PCR. (PDF 167 kb) [file 40478_2018_555_MOESM2_ESM.pdf]

**Table S2: PCR Primers**

For patient samples

| Gene | Forward (5'-3') | Reverse (5'-3') |
|---|---|---|
| *BAG3* | GCACCACTACGTGGAACGA | GGTGGCCTTCCCTAGCAG |
| *CHORDC1* | CCTAAGCCAGTAGAAGCAATAAAAA | TGATGACAGTTTAAGTTTATCAAGTGC |
| *CRYAB* | CAGCTGGTTTGACACTGGAC | GCTTCACATCCAGGTTGACA |
| *DEDD2* | CAGTGCGACGAGAGCAAC | TAGCGTTCTGGAGACACTGG |
| *DNAJB1* | GGCCTACGACGTGCTCAG | GTGTAGCTGAAAGAGGTACCATTG |
| *DNAJB4* | AGGTCGCAGAAGCTTATGAAGT | CCTCCTGCTCCTCCTTTCA |
| *FKBP4* | CGGGAGAAGAAGCTCTATGC | GGTCTCCTGAGGAAGCCTCT |
| *GAPDH* | GTTCGACAGTCAGCCGCATC | GGAATTTGCCATGGGTGGA |
| *HSF1* | CAAGCTGTGGACCCTCGT | TCGAACACGTGGAAGCTGT |
| *HSPA1A* | CGGCAAGGTGGAGATCAT | GGTGTTCTGCGGGTTCAG |
| *HSPA1B* | AAGGGTGTTTCGTTCCCTTT | TAGTGTTTTCGCCAAGCAAA |
| *HSPB1* | TCCCTGGATGTCAACCACTT | GATGTAGCCATGCTCGTCCT |
| *RPLP0* | TCTACAACCCTGAAGTGCTTGAT | CAATCTGCAGACAGACACTGG |
| *SERPINH1* | GGTGGAGGTGACCCATGA | CTTGTCAATGGCCTCAGTCA |
| *STIP1* | CGAAAGATGCCAAATTATACAGC | CTGGATACATTCCTCACAGTCCT |

For neuron experiments

| Gene | Forward (5'-3') | Reverse (5'-3') |
|---|---|---|
| *ACTB* | AGATCAAGATCATTGCTCCTCCT | CGGACTCGTCATACTCCTGC |
| *BAG3* | TGGGAGATCAAGATCGACCC | GGGCCATTGGCAGAGGATG |
| *CHORDC1* | CCTTGCTGTGCTACAACCG | CGGAACACCTGGGTGGTATG |
| *GAPDH* | TTGTCAAGCTCATTTCCTGGTATG | TCCTCTTGTGCTCTTGCTGG |
| *HSF1* | CAAGCTGTGGACCCTCGT | TCGAACACGTGGAAGCTGT |
| *HSPA1B* | TTTGAGGGCATCGACTTCTACA | CCAGGACCAGGTCGTGAATC |
| *HSPA6* | CAAGGTGCGCGTATGCTAC | GCTCATTGATGATCCGCAACAC |
| *SERPINH1* | TCAGTGAGCTTCGCTGATGAC | CATGGCGTTGACTAGCAGGG |
| *STIP1* | CCTTACAGTGCTACTCCGAAGC | ATAGGCAGCAGAACGGTTGC |

For *Drosophila* experiments

| Gene | Forward (5'-3') | Reverse (5'-3') |
|---|---|---|
| *dHSF1* | ATCGCTTGATTTGCTGGACC | GAAGCTGGCCATGTTGTTGT |
| *dHSP70* | GGCACCTGCTGGCAAATT | CGGATAGTGTCGTTGCACTTGT |
| *dBAG3 (Stv)* | GTACGCCTACCTGGACGAGA | CAACTGCACCTGTGTCAACC |
| *dStip1* | TGGCCTAAGGGTTACTCACG | GCATTCGTGGGATCGTACTT |
| *dFkbp4 (Fkbp59)* | GCTCCAAACTACGCTTACGG | TGGTTGGGACTCAAATCCTC |
| *dChord1* | TATTTCCCGAGCAGGACAAC | TTGCATTGCTCACATTCACA |
| *HSP68* | GAAGGCACTCAAGGACGCTAAAATG | CTGAACCTTGGGAATACGAGTG |
| *dHSP90 (HSP83)* | GGACAAGGATGCCAAGAAGAAGAAG | CAGTCGTTGGTCAGGGATTTGTAG |
| *dHSP40* | GAGATCATCAAGCCCACCACAAC | CGGGAAACTTAATGTCGAAGGAGAC |
| *dHSP27* | GGCCACCACAATCAAATGTCAC | CTCCTCGTGCTTCCCCTCTACC |
| *RP49* | TGTCCTTCCAGCTTCAAGATGACCATC | CTTGGGCTTGCGCCATTTGTG |
