# Supplementary figures and images for: Dipeptide repeat proteins activate a heat shock response found in C9ORF72-ALS/FTLD patients
Source: Acta Neuropathol Commun. 2018 Jul 4;6:55. doi: 10.1186/s40478-018-0555-8 (PMC6031111; doi:10.1186/s40478-018-0555-8)

**Figure S5**

**External Eye Quantification Scale for Gmr-Gal4 > (GR)36**

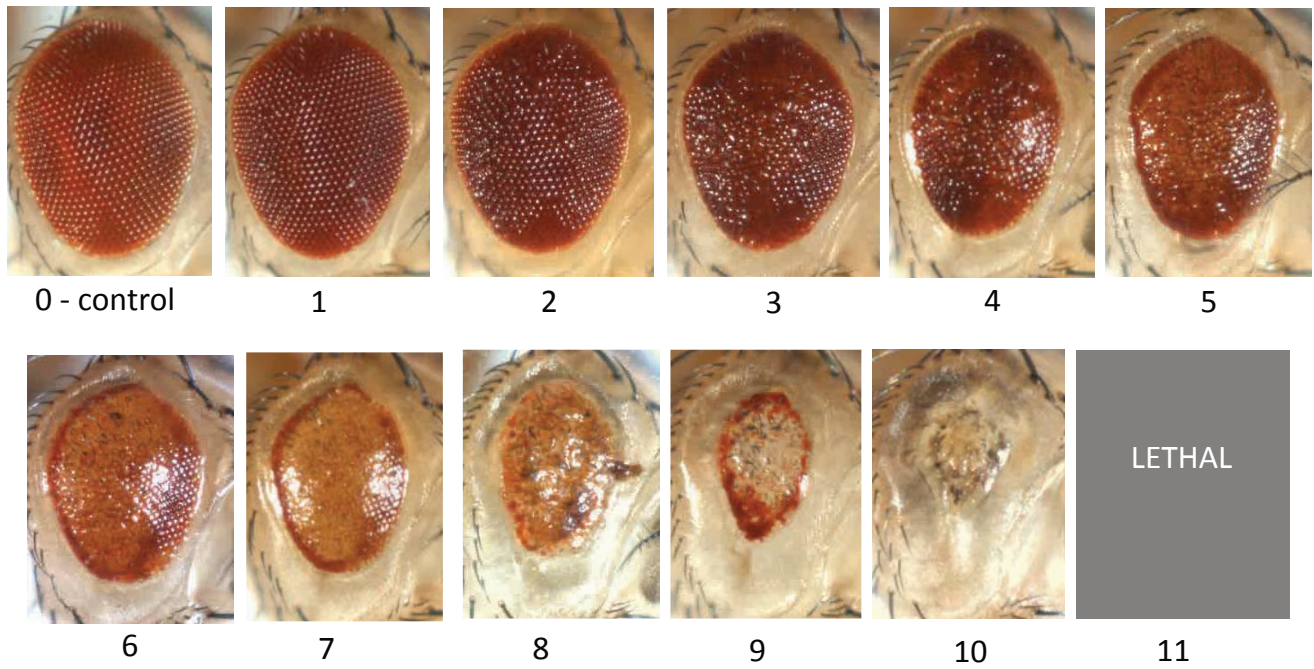

Supplement: Supplementary file 3 — Figure S5. External eye quantification scale for (GR)36 animals. For quantification of the enhancement effects of increased expression of HSF in the eye, (GR)36 animals received a score between 0 (normal eye) and 11 (extreme toxicity causing lethality). Across multiple studies control Gmr-GAL4 > (GR)36 animals receive a score between 5 and 6. (PDF 1969 kb) [file 40478_2018_555_MOESM3_ESM.pdf]

Figure S1

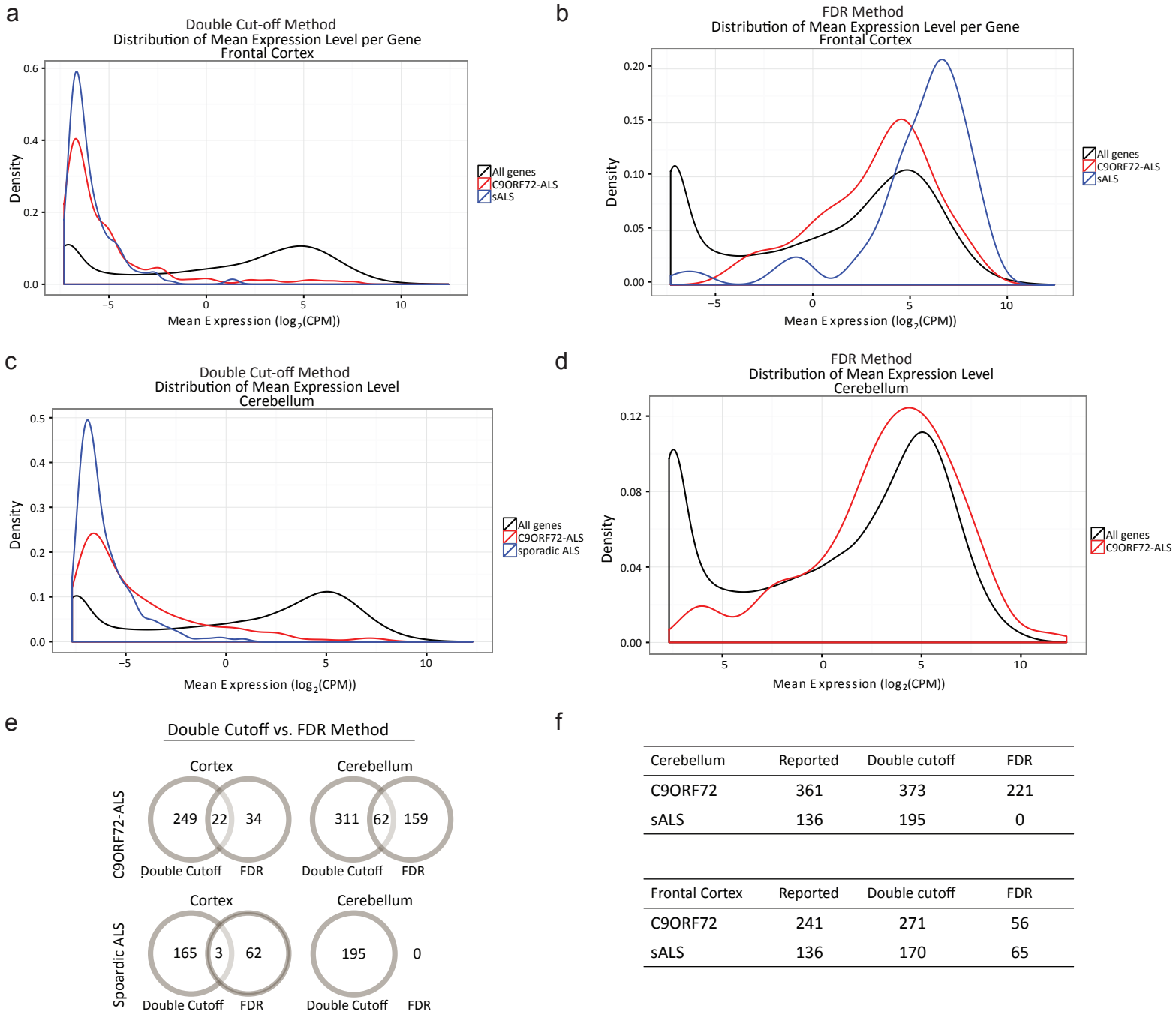

Supplement: Supplementary file 4 — Figure S1. Bioinformatic method comparison for gene expression analysis of C9ORF72-associated ALS and sporadic ALS (sALS) in the frontal cortex and cerebellum. (a-d) Gene density plots comparing the expression levels of differentially expressed transcripts as determined by the prior double cut-off method (|log2 fold change| ≥ 2 and p-value < 0.05) and the FDR method (FDR < 0.05), which was used in Fig. 1 and subsequent analysis. Note, no significant changes were detected in the sALS cerebellum using with FDR method. (e) Venn diagrams demonstrating considerable differences in the designated disease-associated transcripts between these bioinformatics methods in both brain regions. (f) Table of the number of differentially expressed transcripts as determined by Prudencio et al., the double cut-off method and the FDR method used here. (PDF 216 kb) [file 40478_2018_555_MOESM4_ESM.pdf]

Figure S2

a

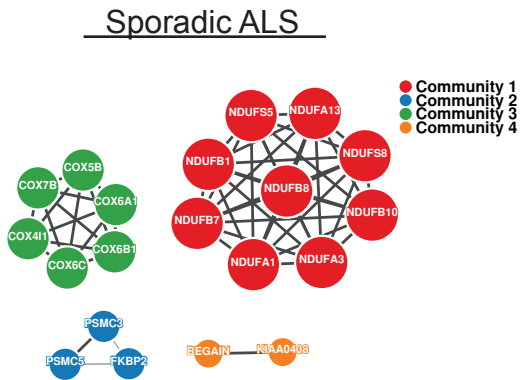

b

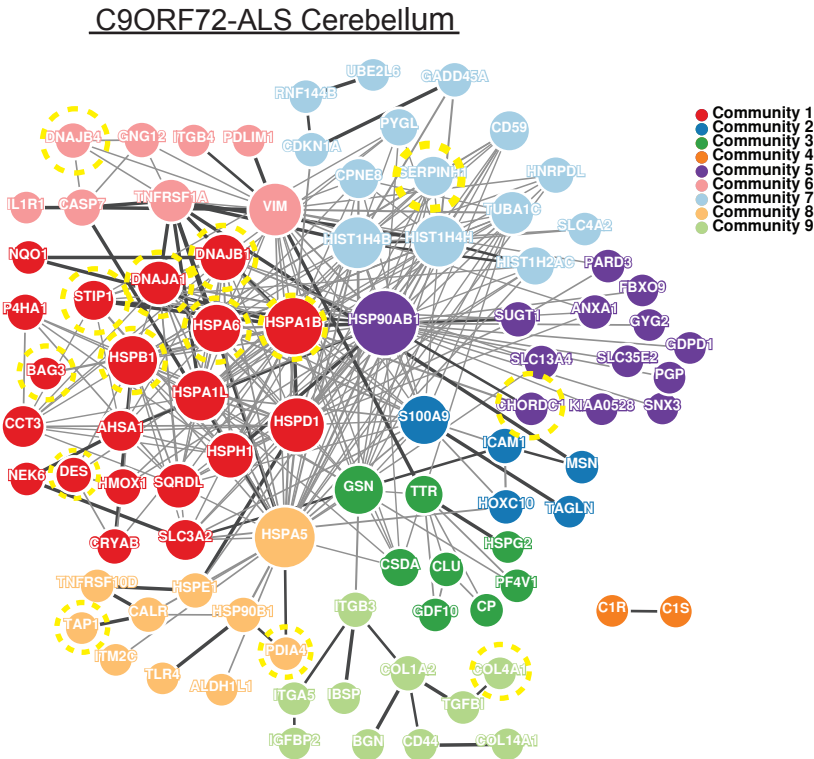

Supplement: Supplementary file 6 — Figure S2. Gene networks in C9ORF72-ALS and sporadic ALS. (a) Protein-protein interaction network derived from differentially expressed transcripts in C9ORF72-ALS cerebellum. Those transcripts that are differentially expressed in both the frontal cortex and the cerebellum in C9ORF72-ALS are highlighted by dashed yellow circles, and predominantly consist of heat shock proteins and protein chaperones. (b) Protein-protein interaction network derived from differentially expressed transcripts in the sporadic ALS cortex. (PDF 164 kb) [file 40478_2018_555_MOESM6_ESM.pdf]

Figure S4

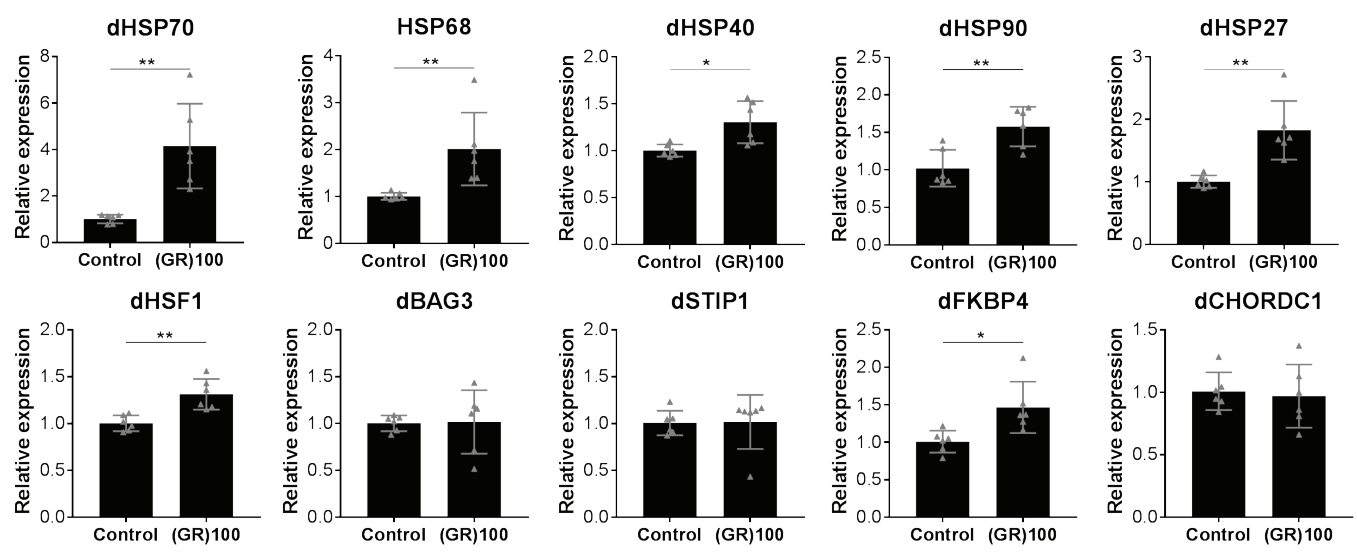

Supplement: Supplementary file 9 — Figure S4. poly-GR expression results in the upregulation of heat shock response genes and dHSF1 in the adult fly nervous system. UAS-(GR)36 was expressed in the adult fly nervous system using the drug-inducible Gal4 driver, elavGS, for 16d. qPCR analysis of endogenous HSF1-regulated genes and dHSF1 revealed significant upregulation of the Drosophila orthologs of many of the genes identified in patient studies, suggesting that poly-GR is contributing to the altered transcriptome in C9ORF72-ALS/FTLD patients. Control animals did not express a transgene. Differences in expression are likely underestimated as the analyses include neuronal and non-neuronal tissue while (GR)36 was expressed only in neurons. (n = 6, mean +/− SD, unpaired, student’s t-tests, p-value * < 0.05, ** < 0.01). (PDF 389 kb) [file 40478_2018_555_MOESM9_ESM.pdf]
