# Supplementary material for: Dipeptide repeat proteins activate a heat shock response found in C9ORF72-ALS/FTLD patients
Source: Acta Neuropathol Commun. 2018 Jul 4;6:55. doi: 10.1186/s40478-018-0555-8 (PMC6031111; doi:10.1186/s40478-018-0555-8)
Supplement: Supplementary file 7 — Table S4. Values from qPCR analysis in brain samples. (PDF 178 kb) [file 40478_2018_555_MOESM7_ESM.pdf]

Frontal Cortex

	controls		C9 minus		C9 plus	
Transcript	Mean	SEM	Mean	SEM	Mean	SEM
BAG3	1	0.1941	2.441	0.3075	6.43	1.133
HSPB1	1	0.1674	2.181	0.2635	4.532	0.5553
DNAJB1	1	0.09156	1.376	0.193	3.685	0.7109
DNAJB4	1	0.09594	0.9647	0.04555	1.35	0.09538
STIP1	1	0.06504	1.006	0.04941	1.364	0.08631
FKBP4	1	0.07378	1.044	0.0667	1.71	0.1495
CRYAB	1	0.1693	1.506	0.17	2.51	0.2583
SERPINH1	1	0.1937	2.147	0.3143	5.362	1.286
DEDD2	1	0.08292	1.084	0.06472	1.884	0.1495
CHORDC1	1	0.1055	1.24	0.103	2.066	0.18
HSPA1A	1	0.1489	1.976	0.3901	4.505	0.7859
HSPA1B	1	0.227	1.841	0.4919	4.456	0.9235
HSF1	1	0.08649	1.153	0.07217	1.559	0.1046

Cerebellum

	controls		C9 minus		C9 plus	
Transcript	Mean	SEM	Mean	SEM	Mean	SEM
BAG3	1	0.1582	1.315	0.1172	4.001	0.7829
HSPB1	1	0.2488	2.597	0.4193	8.004	1.412
DNAJB1	1	0.08901	2.239	0.4234	11.36	2.981
DNAJB4	1	0.1066	1.525	0.1481	2.958	0.3366
STIP1	1	0.06839	1.641	0.1931	3.13	0.3453
FKBP4	1	0.08832	1.51	0.1573	3.066	0.4001
CRYAB	1	0.1176	1.578	0.1246	2.901	0.2779
SERPINH1	1	0.221	2.17	0.4068	9.489	2.65
DEDD2	1	0.1034	1.314	0.1276	3.205	0.4688
CHORDC1	1	0.1522	1.83	0.3235	4.705	0.7268
HSPA1A	1	0.1325	3.39	0.8069	15.41	3.155
HSPA1B	1	0.1606	2.992	0.7493	16.38	3.816
HSF1	1	0.09104	1.19	0.1395	1.708	0.1556

Table S4: Quantitative RT-PCR values for brain samples.
