# Supplementary material for: Dipeptide repeat proteins activate a heat shock response found in C9ORF72-ALS/FTLD patients
Source: Acta Neuropathol Commun. 2018 Jul 4;6:55. doi: 10.1186/s40478-018-0555-8 (PMC6031111; doi:10.1186/s40478-018-0555-8)
Supplement: Supplementary file 8 — Figure S3. Activation of HSF1 in C9ORF72-ALS, FTLD, and combined ALS/FTLD patients. (a) Quantitative real-time PCR (qRT-PCR) for HSF1 target genes in the cerebellum of sporadic and C9ORF72-associated disease (n = 56 C9ORF72-ALS/FTLD, n = 42 sporadic ALS/FTLD, n = 7 controls) (one-way ANOVA with Bonferonni post-hoc test for multiple comparisons * p < 0.05, ** p < 0.01, *** p < 0.001. Note, no significant changes were detected between the sporadic cases and controls. (b) Correlation of HSF1 levels and HSF1 target gene levels in the frontal cortex and cerebellum in C9ORF72-ALS/FTLD. Spearman’s R2 values are plotted for each target gene, error bars denote 95% confidence interval, p-value < 0.0001 in all cases. (PDF 209 kb) [file 40478_2018_555_MOESM8_ESM.pdf]

Figure S3

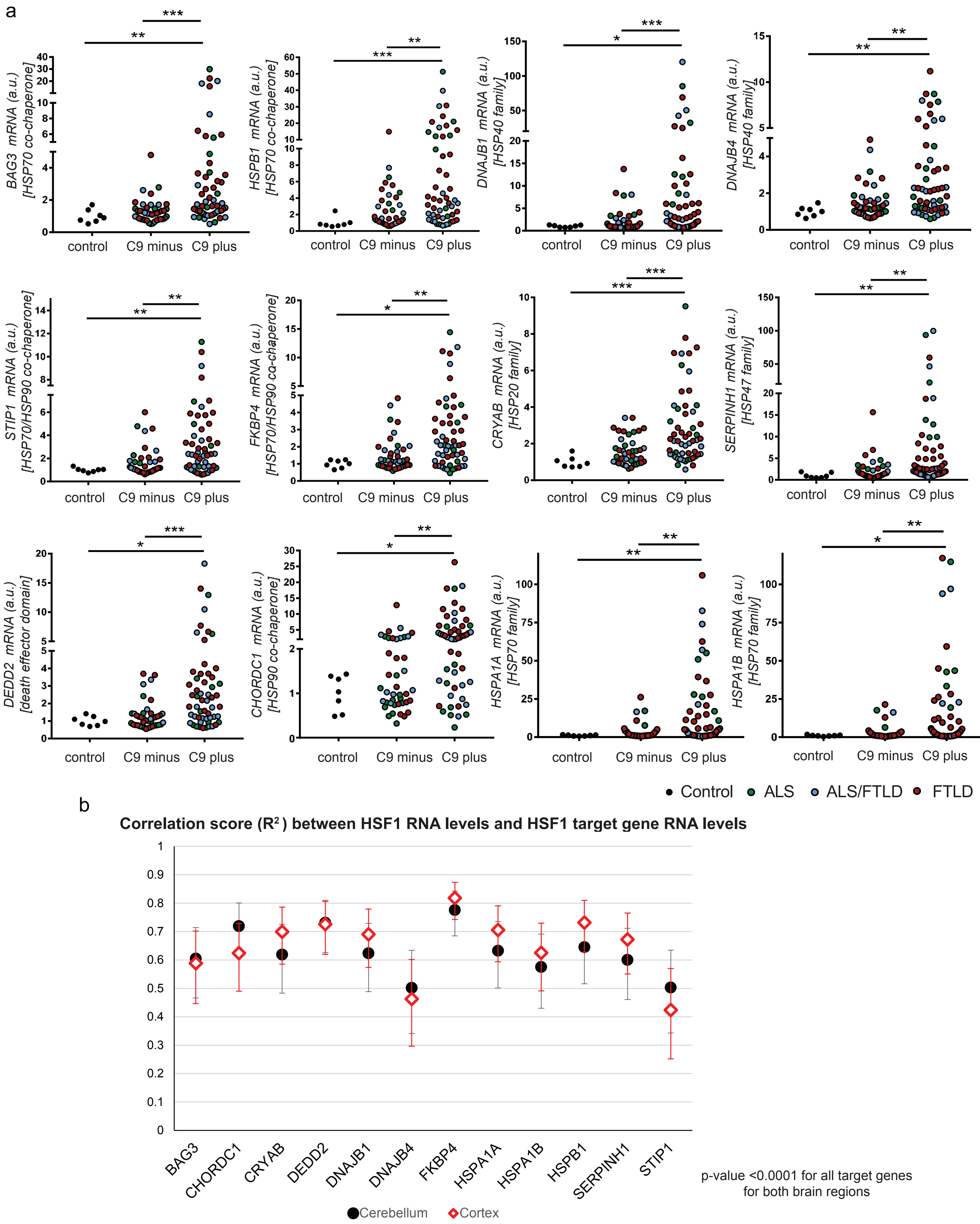
a
BAG3 mRNA (a.u.) [HSP70 co-chaperone]
HSPB1 mRNA (a.u.) [HSP70 co-chaperone]
DNAJB1 mRNA (a.u.) [HSP40 family]
DNAJB4 mRNA (a.u.) [HSP40 family]
STIP1 mRNA (a.u.) [HSP70/HSP90 co-chaperone]
FKBP4 mRNA (a.u.) [HSP70/HSP90 co-chaperone]
CRYAB mRNA (a.u.) [HSP20 family]
SERPINH1 mRNA (a.u.) [HSP47 family]
DEDD2 mRNA (a.u.) [death effector domain]
CHORDC1 mRNA (a.u.) [HSP90 co-chaperone]
HSPA1A mRNA (a.u.) [HSP70 family]
HSPA1B mRNA (a.u.) [HSP70 family]
control
C9 minus
C9 plus
Control
ALS
ALS/FTLD
FTLD
b
Correlation score (R²) between HSF1 RNA levels and HSF1 target gene RNA levels
BAG3
CHORDC1
CRYAB
DEDD2
DNAJB1
DNAJB4
FKBP4
HSPA1A
HSPA1B
HSPB1
SERPINH1
STIP1
p-value <0.0001 for all target genes for both brain regions
Cerebellum
Cortex
